# Supplementary material for: The Potential of PSMA as a Vascular Target in TNBC
Source: Cells. 2023 Feb 8;12(4):551. doi: 10.3390/cells12040551 (PMC9954547; doi:10.3390/cells12040551)
Supplement: Supplementary file 1 [file cells-12-00551-s001.zip › cells-2055133-supplementary.pdf]

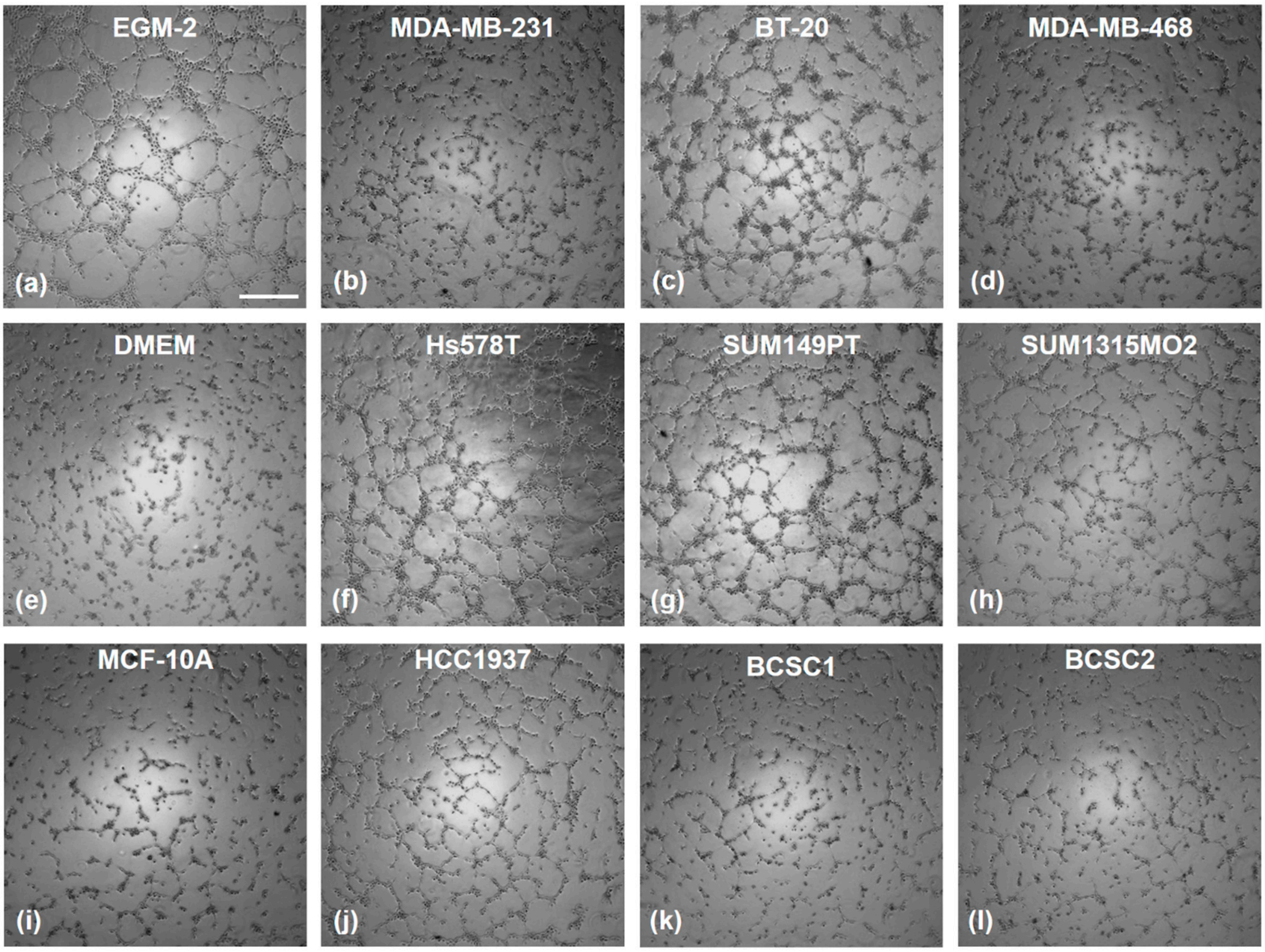

Figure S1. Tube formation assay in HUVEC. Phase contrast images of HUVEC tube formation incubated in the indicated TNBC TCM were acquired after 5 h. EGM-2 (+) and DMEM (−) served as control media. Scale bar: 500 μm.

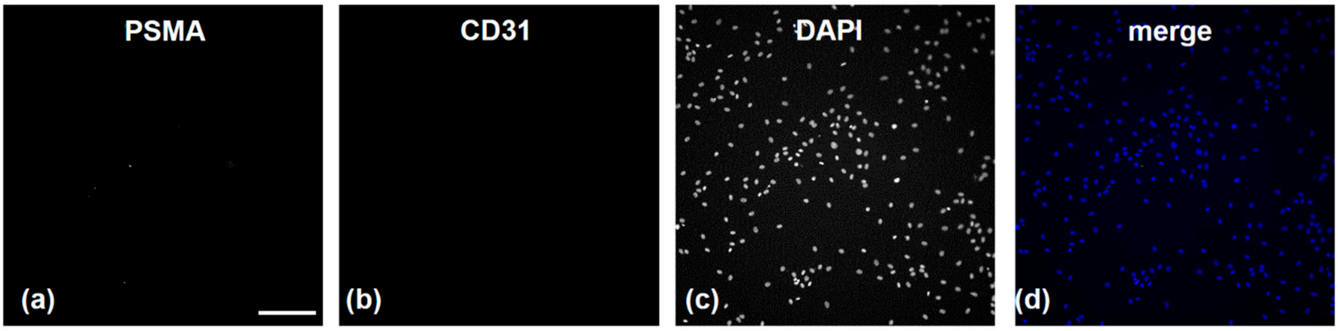

Figure S2. Antibody control. Secondary antibodies Alexa Fluor 488 and 555 were applied without primary antibodies to exclude unspecific binding. Nuclei were stained with DAPI. Scale bar: 200 μm.

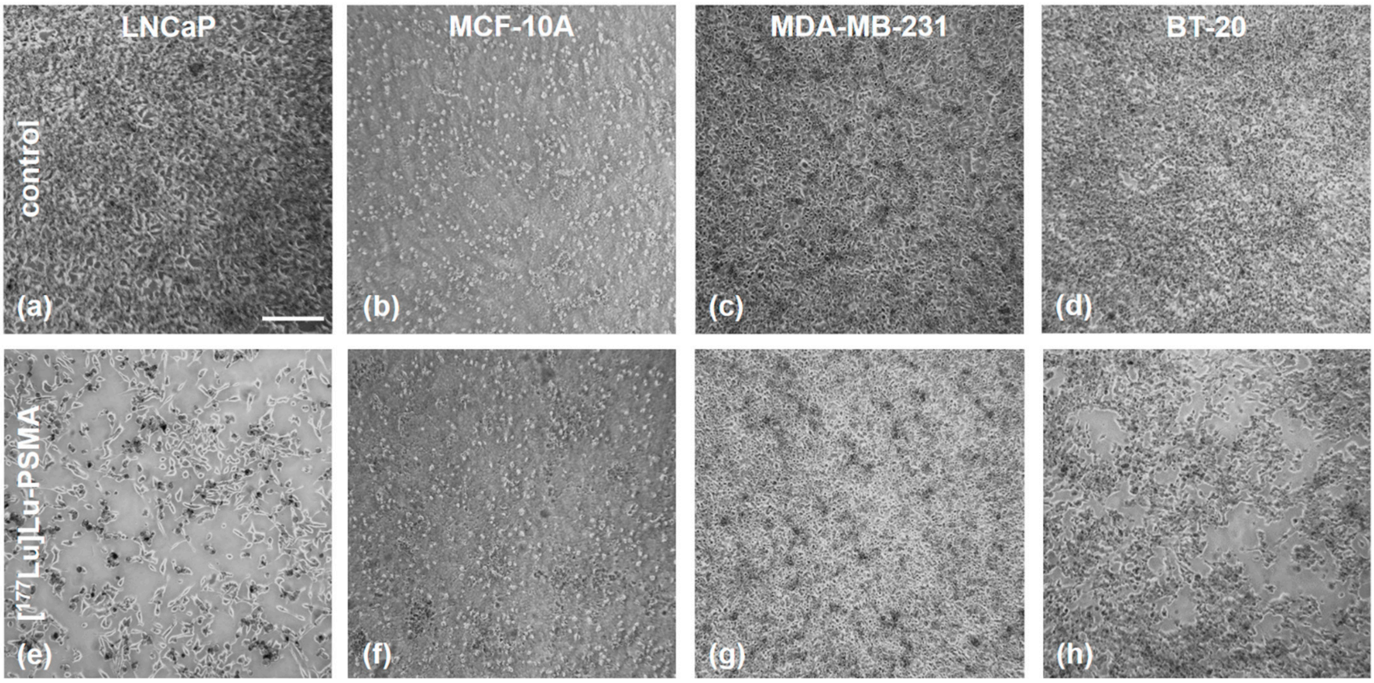

Figure S3. Cell viability of TNBC cells after treatment with [^{177}Lu]Lu-PSMA. Phase contrast images of the indicated cell lines were acquired subsequently before flow cytometry analysis. Scale bar: 500 μm.

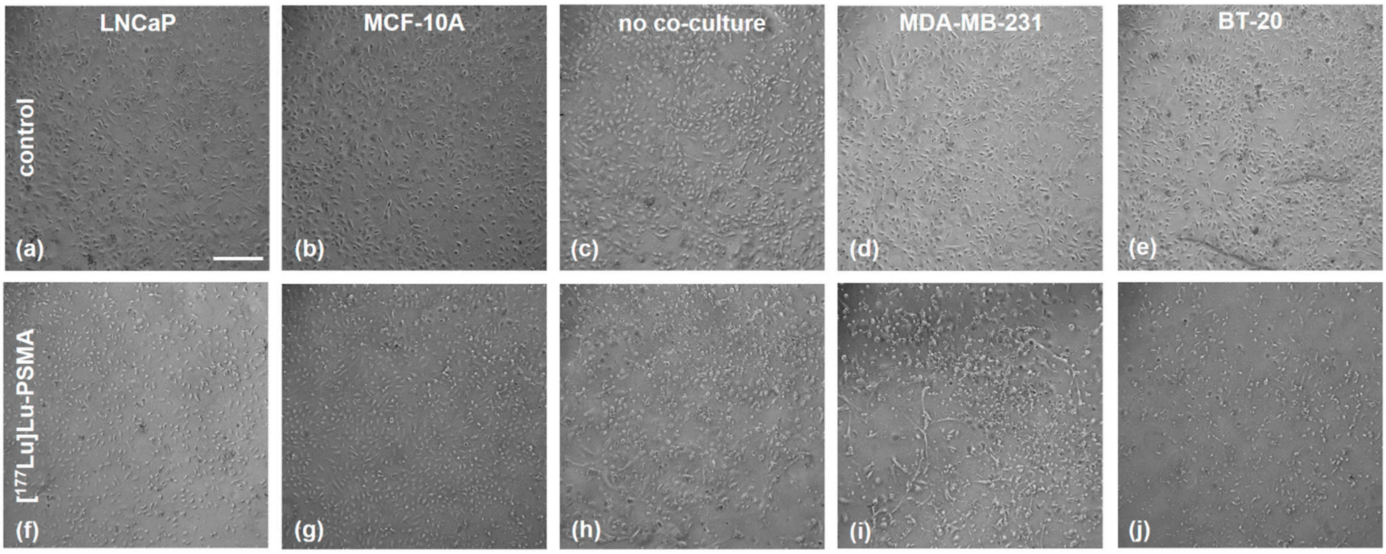

Figure S4. Cell viability of HUVECs co-cultured with the indicated cell lines after treatment with [^{177}Lu]Lu-PSMA. Phase contrast images of the indicated cell lines were acquired subsequently before flow cytometry analysis. Scale bar: 500 μm.
